# Supplementary material for: Origin of high thermal conductivity in disentangled ultra-high molecular weight polyethylene films: ballistic phonons within enlarged crystals
Source: Nat Commun. 2022 May 4;13:2452. doi: 10.1038/s41467-022-29904-2 (PMC9068786; doi:10.1038/s41467-022-29904-2)
Supplement: Supplementary file 1 — Supplementary information as PDF [file 41467_2022_29904_MOESM1_ESM.pdf]

Supporting information: Origin of high thermal conductivity in disentangled ultra-high molecular weight polyethylene films: ballistic phonons within enlarged crystals

Taeyong Kim,[1] Stavros X. Drakopoulos,[2] Sara Ronca,[2] and Austin J. Minnich[1,*]

[1]*Division of Engineering and Applied Science, California Institute of Technology, Pasadena, California 91125, USA*

[2]*Department of Materials, Loughborough University, Loughborough LE11 3TU, United Kingdom*

(Dated: April 4, 2022)

* aminnich@caltech.edu

SI. ADDITIONAL TRANSIENT GRATING DATA

Additional TG data for various grating periods and temperatures are presented below. In the fitted curves, oscillations appear at nanosecond time scales due to the impulse response of the detector. To facilitate comparing the predicted and measured TG signal, we computed the fitting curves using a convolution between the detector's measured impulse response and the exponential decay corresponding to the material response. Therefore, oscillations due to the impulse response of the detector appear at time scales around the inverse bandwidth of the detector.

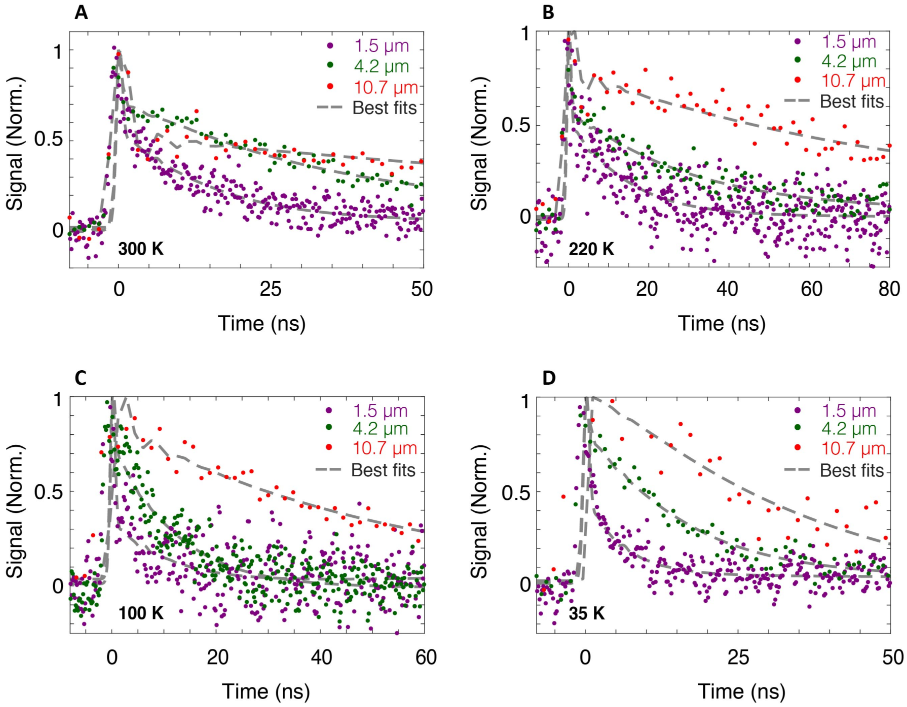

Figure SI. Additional TG measurements and corresponding best fits for grating periods of 1.5 μm, 4.2 μm, and 10.7 μm at (A) 300 K, (B) 220 K, (C) 100 K, and (D) 35 K.

SII. THERMAL CONDUCTIVITY VERSUS GRATING PERIOD

Additional measurements of thermal conductivity versus grating period at 220 K and 35 K are shown in Fig. SII along with the model predictions. The model captures the general trend although quantitative discrepancies remain.

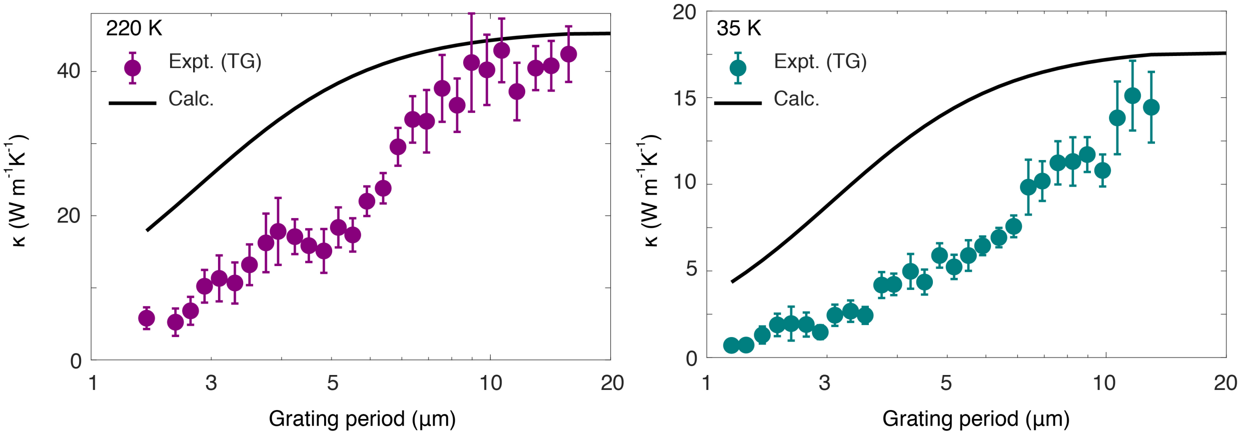

Figure SII. Measured thermal conductivity versus grating period along with the fit based on an anistropic Debye model at (A) 220 K and (B) 35 K.

SIII. COMPUTED THERMAL CONDUCTIVITY VERSUS GRATING PERIOD VERSUS TEMPERATURE USING OTHER CANDIDATE PROFILES

This section presents calculated thermal conductivity using alternate trends of MFP for the LA branch relaxation time versus frequency. MFP profiles were constructed using Matthiessen's rule as described in the main text, except with different frequency power laws (either $n = 1, 2$ in this section versus $n = 4$ in the main text). The first trend is a constant MFP (Λ_0) of 340 nm that transitions to ω^{-1} at 7 THz, shown in Fig. SIII; the second is a constant MFP of 340 nm that transitions to ω^{-2} at 7 THz, shown in Fig. SIV. Power laws with larger exponents generally exhibit improved agreement of thermal conductivity versus grating period.

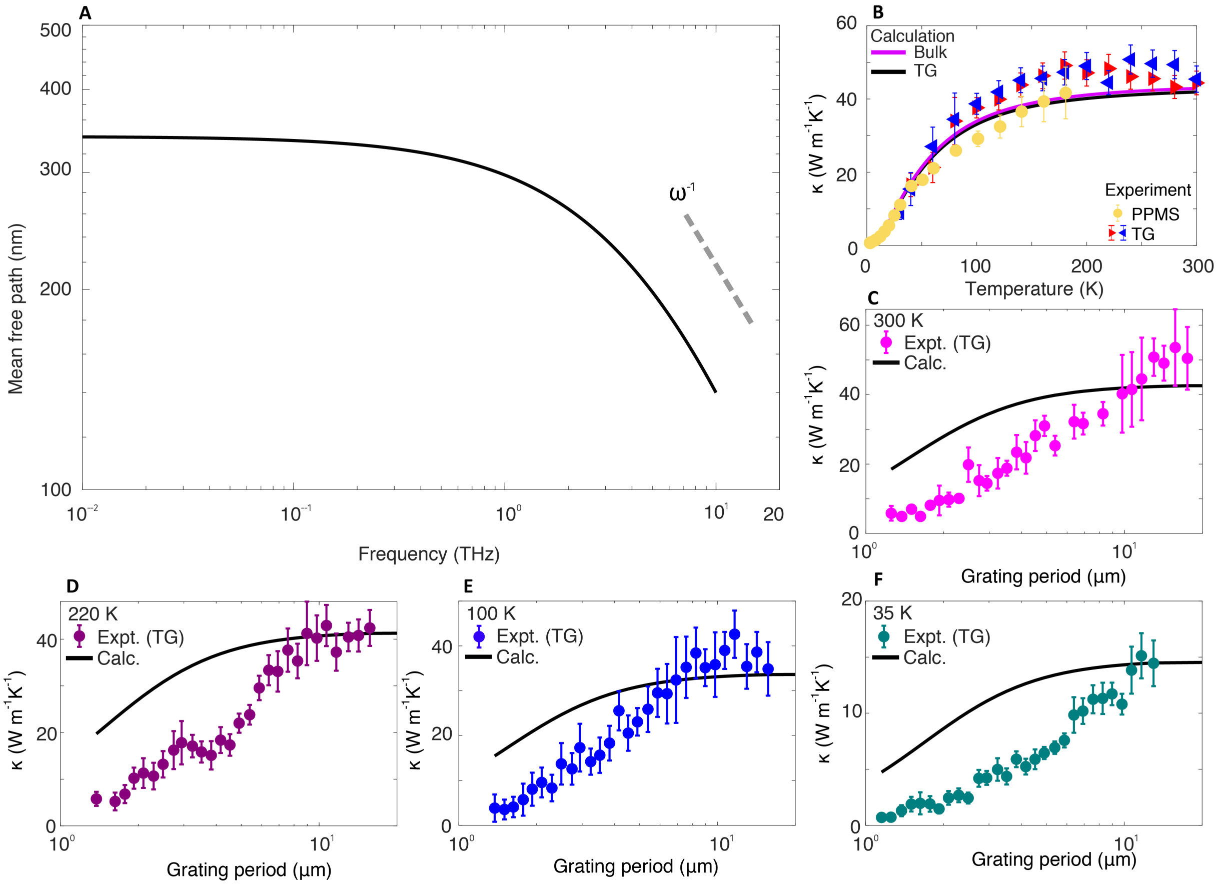

Figure SIII. (A) Candidate MFP profile versus frequency (Constant value (340 nm) to ω^{-1}, transition frequency $\sim$ 7 THz). (B) Calculated bulk thermal conductivity versus temperature using the profile in (A). Calculated thermal conductivity versus grating period at (C) 300 K, (D) 220 K, (E) 100 K, and (F) 35 K using the profile in (A).

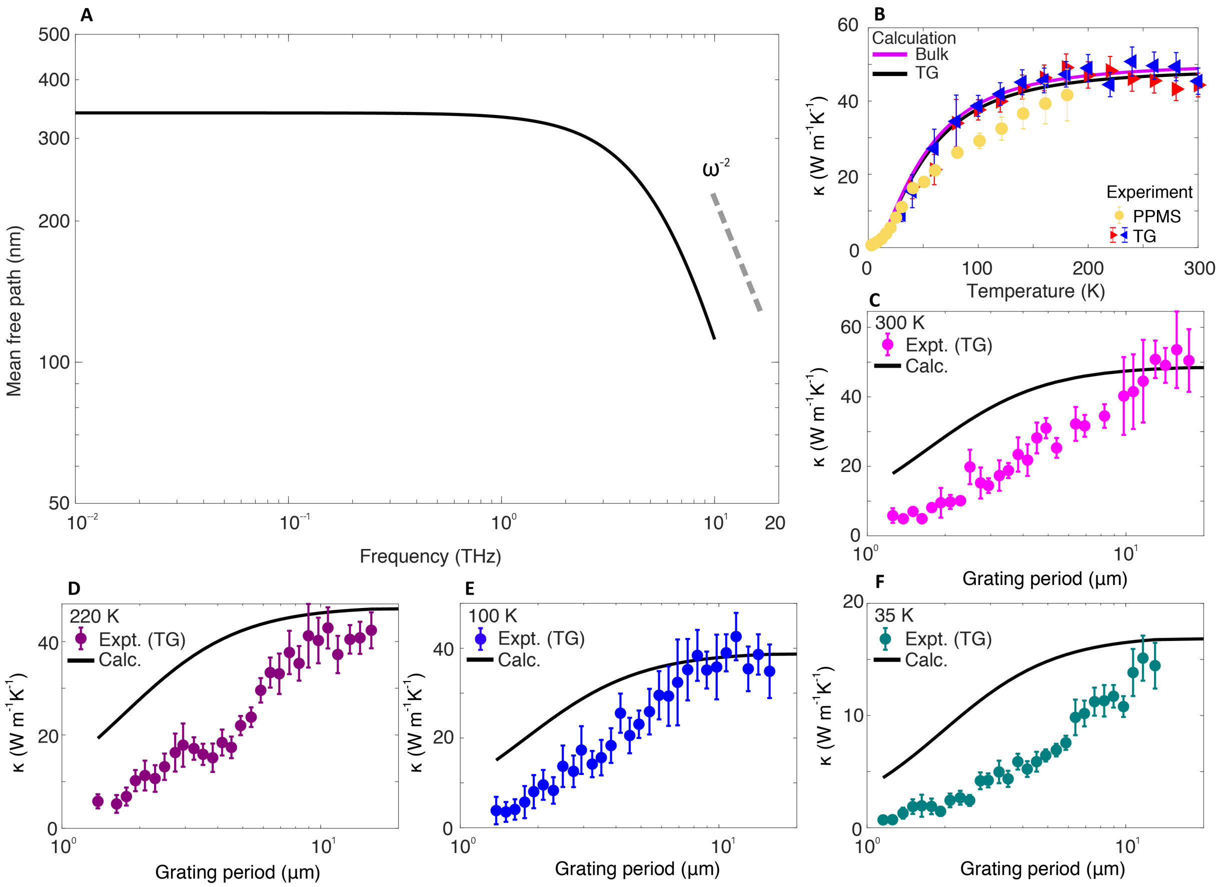

Figure SIV. (A) Candidate MFP profile versus frequency (Constant value (340 nm) to ω^{-2}, transition frequency $\sim$ 7 THz). (B) Calculated bulk thermal conductivity versus temperature using the profile in (A). Calculated thermal conductivity versus grating period at (C) 300 K, (D) 220 K, (E) 100 K, and (F) 35 K using the profile in (A).

SIV. DETAILS OF THE PHYSICAL PROPERTY MEASUREMENT SYSTEM MEASUREMENTS

PPMS measurements were conducted at temperatures ranging from $\sim$ 3 - 200 K, and a detailed description of the experimental geometry is described in the Methods section in the main text. At each temperature, we adjusted the electrical heater power to minimize the temperature rise and temperature fluctuations during thermal equilibration. Table SI shows the base temperature and peak temperature along with the electrical power. The temperature rise was constrained to be 1.3 - 3.5% of the base temperature. To account for radiative heat loss, we used an emissivity of 0.2 as given in Ref. [1].

Table SI. Measured sample temperature and electrical power used in PPMS.

Base sample temperature (K)	Heater power (μW)	Peak temperature (K)
3	0.3	3.0
3	0.5	3.1
4	0.6	4.1
8	4	8.3
12	8	12.4
16	20	16.7
20	30	20.7
25	50	25.8
30	120	31.5
41	240	42.0
50	320	52.4
61	400	62.5
81	700	83.5
101	950	104.1
121	1.2×10^3	124.4
141	1.05×10^3	143.2
161	1.45×10^3	163.9
181	1.48×10^3	183.5

SV. STEADY HEATING AT 30 K

We estimate the steady heating due to the pump and probe pulses at 30 K. At higher temperatures, the thermal conductivity of the sample is high enough that the steady temperature rise relative to the base temperature can be neglected. Given the laser beam diameter $d = 550$ μm, and heat conduction length $l = 10$ mm (see Fig. 1B), the steady temperature rise considering 1D heat conduction along the drawing direction can be expressed as

$$\Delta T = \frac{l}{A}\frac{P_{abs,total}}{\kappa} \tag{S1}$$

where $A = dt \simeq 550$ μm $\times 30$ μm $= 1.7 \times 10^{-8}$ m^2 is the cross-sectional area, $P_{abs,total} = \alpha P_{total}$ is the absorbed average power with incident optical power P_{total}, and κ is the thermal conductivity at 30 K (~ 10 $Wm^{-1}K^{-1}$). P_{total} was calculated to be ~ 1.75 mW, using $P_{total} = (1/2)(E_{pump} \times f_{rep} + P_{probe})$ where E_{pump} is incident pump energy (~ 13 μJ), f_{rep} is laser repetition rate (200 Hz), and P_{probe} is the steady incident probe power (≈ 900 μW). The factor of 1/2 accounts for heat conduction in both directions from the center of the film to the edges. Since experimentally determining α of the sample is challenging due to intense optical scattering of the sample, α was roughly estimated as $\sim 5\%$. The resulting steady temperature rise is estimated to be $\Delta T \sim 10$ mm $/(1.7 \times 10^{-8}$ m$^2)$ $\times(1.75$ mW $\times 0.05/10$ Wm^{-1}K$^{-1}) \sim 5.3$ K. Therefore, we take the temperature of the sample for a base temperature of 30 K to be ~ 35 K.

SVI. SURFACE CHARACTERIZATION USING ATOMIC FORCE MICROSCOPY (AFM)

This section provides the surface profile measured from atomic force microscopy (AFM). The AFM topography is shown in Fig. SV. The AFM crosscut perpendicular to the fiber alignment direction (dashed line in Fig. SV(A)) is shown in Fig. SV(B). The calculated RMS roughness is ~ 70 nm, and the maximum peak-to-valley difference is ~ 360 nm. The height difference indicates the surface inhomogeneity over length scales comparable to the optical wavelength (515 nm) used, explaining the intense optical scattering observed in the TG experiment.

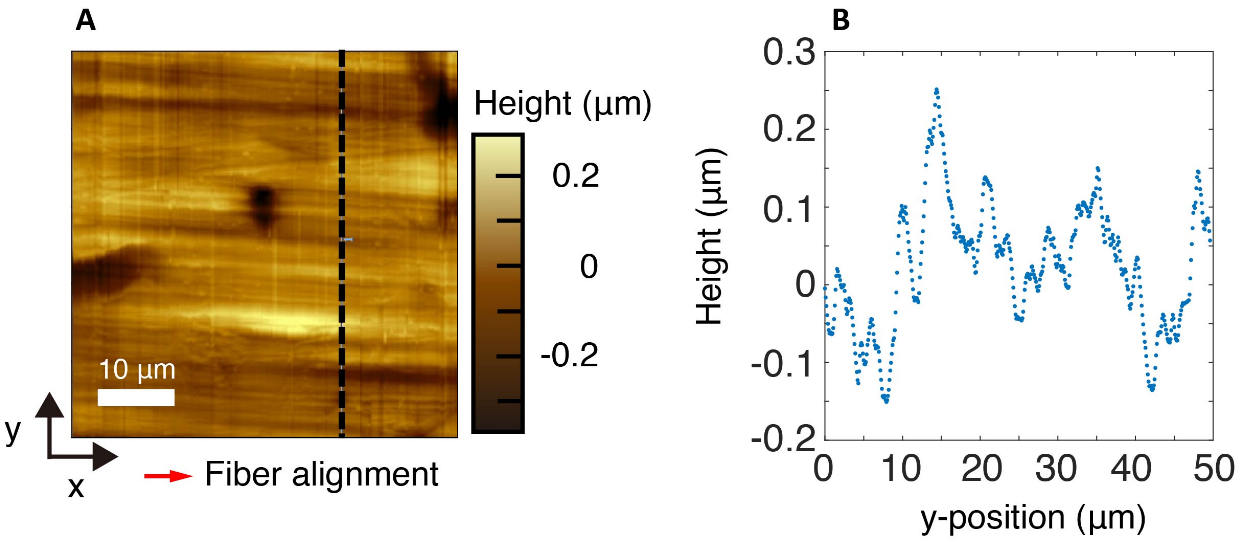

Figure SV. AFM Characterization of the UHMWPE surface. A. AFM topographic image of the sample. The x- and y- direction indicates parallel (perpendicular) to the fiber alignment. B. AFM crosscut along the dashed line in (A). RMS roughness is calculated to be ~ 70 nm, and the maximum peak-to-valley difference is ~ 360 nm.

REFERENCES

[1] Yanfei Xu, Daniel Kraemer, Bai Song, Zhang Jiang, Jiawei Zhou, James Loomis, Jianjian Wang, Mingda Li, Hadi Ghasemi, Xiaopeng Huang, Xiaobo Li, and Gang Chen. Nanostructured polymer films with metal-like thermal conductivity. *Nature Communications*, 10(1):1–8, April 2019. ISSN 2041-1723. doi: 10.1038/s41467-019-09697-7. URL https://www.nature.com/articles/s41467-019-09697-7.
